# Controlled Delivery of Zoledronate Improved Bone Formation Locally *In Vivo*

**DOI:** 10.1371/journal.pone.0091317

**Published:** 2014-03-11

**Authors:** Wenlong Gou, Xin Wang, Jiang Peng, Qiang Lu, Yu Wang, Aiyuan Wang, Quanyi Guo, Xupeng Gao, Wenjing Xu, Shibi Lu

**Affiliations:** Instistute of Orthopaedics, Chinese PLA General Hospital, Haidian District, Beijing, P.R. China; INSERM U1059/LBTO, Université Jean Monnet, France

## Abstract

Bisphosphonates (BPs) have been widely used in clinical treatment of bone diseases with increased bone resorption because of their strong affinity for bone and their inhibition of bone resorption. Recently, there has been growing interest in their improvement of bone formation. However, the effect of local controlled delivery of BPs is unclear. We used polylactide acid-glycolic acid copolymer (PLGA) as a drug carrier to deliver various doses of the bisphosphonate zoledronate (Zol) into the distal femur of 8-week-old Sprague-Dawley rats. After 6 weeks, samples were harvested and analyzed by micro-CT and histology. The average bone mineral density and mineralized bone volume fraction were higher with medium- and high-dose PLGA-Zol (30 and 300 µg Zol, respectively) than control and low-dose Zol (3 µg PLGA-Zol; p<0.05). Local controlled delivery of Zol decreased the numbers of osteoclast and increased the numbers of osteoblast. Moreover, local controlled delivery of medium- and high-dose Zol accelerated the expression of bone-formation markers. PLGA used as a drug carrier for controlled delivery of Zol may promote local bone formation.

## Introduction

The skeletal system functions and is maintained on the basis of the balance between osteoclast-mediated bone resorption and osteoblast-mediated bone formation. Bone fragility leading to fracture and disability is implicated in the pathogenesis of various bone resorption diseases. Preservation of bone strength, mainly determined by the amount of minerals, is key to the management of these conditions [1].

Bisphosphonates (BPs) are synthetic analogs of naturally occurring pyrophosphate (P-O-P) in which a carbon atom replaced the oxygen atom connecting the two phosphates (P-C-P) [2]. Because of their strong affinity for bone and their inhibition of bone resorption, BPs have been widely used in clinical treatment of bone diseases with increased bone resorption, including Paget's disease, hypercalcemia of malignancy, fibrous dysplasia, and inflammation-related bone loss [3]–[7]. Recent studies indicated that BPs not only inhibit bone resorption induced by osteoclasts but also accelerate bone formation induced by osteoblasts, albeit with varying or conflicting effects, in relation to the concentration of BPs [1], [8]–[11]. In most studies, BPs were administered intravenously or orally. However, systemic administration of BPs, especially in high doses and over the long term, increased the risk of osteonecrosis of the jaw [12], [13]. Local administration, such as local injection and implanting allografts soaked with BPs solution, has been used in animal research for the treatment of ischemic osteonecrosis of the femoral head and protecting newly formed bone against resorption [14], [15]. However, to this day, controlled local delivery of BPs has rarely been reported.

Polylactide acid-glycolic acid copolymer (PLGA) is a poly (α-hydroxy-ester) that is depolymerized in the presence of water. Because of biodegradable and bioabsorbable qualities that allow for the passive degradation of the polymer in aqueous environments [16], PLGA has been widely used in the research into controlled release drug delivery [17]–[19]. Previous studies have successfully conjugated alendronate sodium to PLGA [20].

In this study, we used PLGA as a drug carrier to deliver the BP zoledronate (Zol) and investigated whether the PLGA-Zol composite could deliver the Zol gradually, inhibit bone resorption, and promote local bone formation.

## Materials and Methods

### Preparation of PLGA-Zol composite cylinders

PLGA (D,L-lactide 75: glycolide 25) with average molecular weight 10,000 was purchased from the Shandong Institute of Medical Instruments (Jinan, Shandong, China). Zol was purchased from Sigma (SML0223, St. Louis, MO, USA). Details on the synthesis of the polymer conjugate have been reported elsewhere [20]. In brief, various weights of Zol were dissolved in 10% aqueous acetic acid and lyophilized to obtain the free acid form (Edward Modulyo freeze-dryer). The free acid form was dissolved in dimethyl sulphoxide (DMSO) and added to a certain amount of PLGA, previously activated by N^/^-(3-dimethylaminopropyl)-N-ethyl carbodiimide hydrochloride (EDAC). After the completion of the conjugation reaction, the reaction solution was purified by dialysis against 3×11 water (CelluSep H1 MWCO 2000; M-Medical s.r.l., Cornaredo, Italy) and the dialyzed sample was frozen in liquid nitrogen and lyophilized. The composite was melted at 85°C and processed into cylinders (2×4 mm diameter) under the closed condition. The content of Zol in the cylinders was designated as 0, 3, 30 and 300 µg per each sample.

### Ethics statement

This study was carried out in strict accordance with the recommendations in the Guide for the Care and Use of Laboratory Animals of the National Institutes of Health (NIH publication No. 85–23, revised 1985). The protocol was approved by the Animal Care and Use Committee of the Chinese People's Liberation Army General Hospital (Permit Number: 2013-35). All surgery was performed under chloral hydrate anesthesia, and all efforts were made to minimize suffering.

### Animal care and surgery

**Figure 1:**
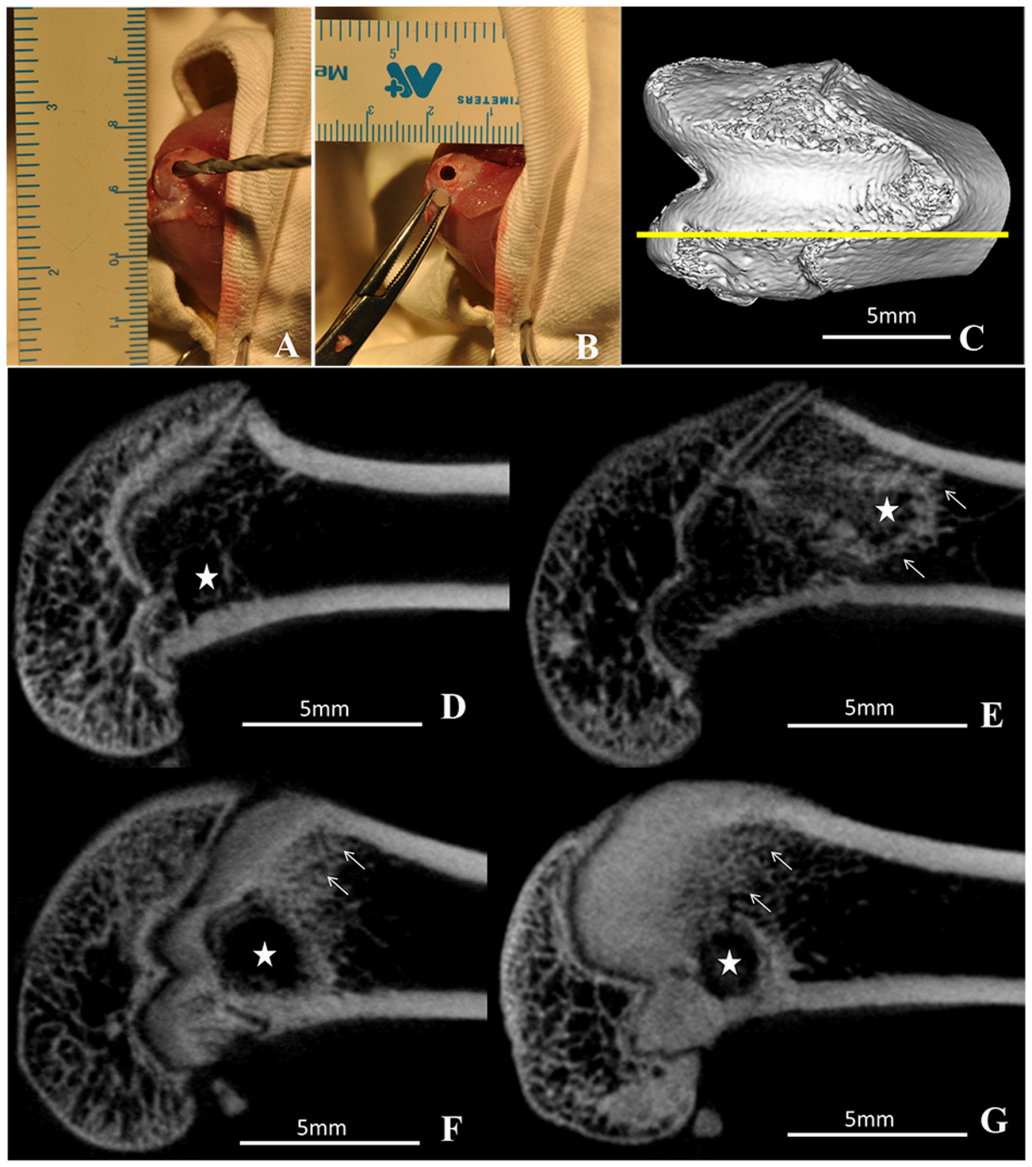
Intraoperative photographs and two-dimensional slices of micro-CT images representing the distal femur. (A) drilling a tunnel (2×4 mm diameter) in the medial femoral condyle along the transepicondylar axis; (B) implantation the PLGA-Zol cylinder; (C) orientation of two-dimensional slice indicated by the yellow line; (D) polylactide acid-glycolic acid copolymer (PLGA) implantation, 0 µg Zol; (E) PLGA-zoledronate (PLGA-Zol), 3 µg Zol; (F) PLGA-Zol, 30 µg Zol; and (G) PLGA-Zol, 300 µg Zol. Stars indicated the site of implantation and arrows indicated where the significant changes happened.

We purchased 40 male Sprague-Dawley rats 8 weeks old (200±25 g) from the Animal Resources Centre (Chinese People's Liberation Army General Hospital, Beijing). Rats were allowed to acclimatize for 1 week to local vivarium conditions before surgery. Animals were housed in a light- and temperature-controlled environment and given food and water ad libitum. Rats were anesthetized with an intraperitoneal injection of 10% chloral hydrate (3.0 ml/kg, Drug Manufacturing Room of CPLA, Beijing), then injected with a preoperative dose of benzyl penicillin (4×10^5^ IU/kg; North China Pharmaceutical Co., Shi Jiazhuang, China) for infection prophylaxis. Under aseptic conditions, a longitudinal incision was made over the anteromedial aspect of the right knee to expose the vertex of the medial femoral condyle. A tunnel (2×4 mm diameter) was made with a drill along the transepicondylar axis, and the PLGA-Zol cylinder (2×4 mm diameter) was implanted into the tunnel (Figure 1A and Figure 1B). After the procedure, rats were given an intramuscular injection of an analgesic (0.05 mg/kg buprenorphine) and allowed unrestricted cage activity.

Rats were randomly divided into 4 groups (10 rats per group) for treatment: 1)control (PLGA cylinder without Zol); 2)low-dose PLGA-Zol (3 µg Zol); 3)medium-dose PLGA-Zol (30 µg Zol); 4)high-dose PLGA-Zol (300 µg Zol).

### Micro-CT evaluation

At 6 weeks, rats were killed by CO_2_ asphyxiation unless otherwise noted. The distal femur was harvested and fixed in 4% formaldehyde. The specimens were scanned by micro-CT (system: RS-9, software: MicroView2.1, GE Healthcare, London, ON, Canada) at 27 µm resolution. The scanning protocol was 80 kV and 450 µA, with an isotopic resolution of 20.589×20.589×20.589 µm voxel size and an integration time of 88 ms. A standardized region of interest (ROI) covering an 8-mm region from the epiphysis to the metaphysis was defined in specimens. Bone mineral density (BMD), mineralized volume fraction (BV/TV), trabecula number (Tb.N), trabecula thickness (Tb.Th.), and trabecula space (Tb.Sp.) were calculated as previously reported [21].

### Histological and histomorphometric analyses

After micro-CT scanning, specimens were decalcified, dehydrated, embedded in paraffin and cut parallel to the long axis of the femur with use of a microtome. Sections from the middle of specimens, each cut at 5-µm intervals, were stained with hematoxylin and eosin (H&E) and Masson trichrome. Osteoclasts and osteoblasts were identified based on both morphology and staining with tartrate resistant acid phosphatase (TRAP) (Sigma, St. Louis, MO, USA)or alkaline phosphatase (modified Gormori staining). Osteoclast and osteoblast numbers were quantitated in the entire area of the bone marrow cavity 1–2 mm from the growth plates and expressed as numbers per square millimeter. Values were calculated from at least five nonconsecutive sections per rat.

### Immunohistochemistry

For immunostaining, commercially available antibodies were used with paraffin-embedded sections for detecting osteocalcin (OCN), bone morphogenetic protein 2 (BMP2), bone morphogenetic protein 7 (BMP7), type I collagen (Col I) and Runt-related transcription factor 2 (RUNX2). The OCN antibody was from Santa Cruz Biotechnology (Santa Cruz. Europe) and others were from Abcam (Hong Kong). Sections were incubated with primary antibodies at a dilution recommended by the manufacturer in a humidified chamber at 4°C overnight, then with 50 µl secondary antibody (Maxim Ltd., Fuzhou, China) for 15 min followed by DAB development. Finally, sections were counterstained with Mayer's hematoxylin for identifying nuclei and mounted with Permount. The negative control was with the same procedure but omitting the primary antibody. Photomicrographs were taken with use of a BX51 Olympus microscope coupled to a DP71 camera (Olympus, Tokyo, Japan) and Picture Frame software. Analysis of images involved ImagePro Plus 5.02 (Media Cybernetics). The special macro for optical density analysis of immunohistochemical staining was acquired at the official web address of the Media Cybernetics Corporation. Using the special macro, the average optical density (AOD) of positive staining area per specimen point in the microarray was calculated as a protein. To eliminate variation, the microscope light source intensity used during image capture was kept constant for all tissue points stained on a given day [22].

### Statistics

All data are presented as mean±standard deviation. Statistical differences among groups were evaluated by Student-Newman-Keuls test (SNK-q) or Mann-Whitney U-test if necessary. Statistical analysis involved use of SPSS 16.0 for Windows (SPSS Inc., Chicago, IL). P<0.05 was considered statistically significant.

## Results

No wound infections occurred in rats. A femoral condyle fracture occurred in a control-group rat during surgery, and it was excluded from the analysis.

### Mineralized tissue formation with PLGA-Zol

**Figure 2:**
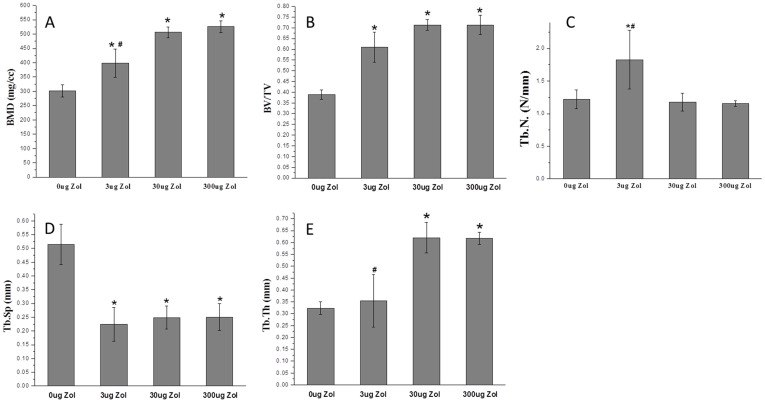
Quantitative micro-CT measurements of (A) bone mineral density (BMD), (B) bone volume fraction (BV/TV), (C) trabecula number (Tb. N.), (D) trabecula thickness (Tb. Th.) and (E) trabecula space (Tb. Sp.). *P<0.05 compared with control group; #P<0.05 compared with PLGA-30 µg Zol and PLGA-300 µg Zol.

Micro-CT revealed a large amount of mineralized tissue, mainly near the distal femoral epiphyseal plate, with medium- and high-dose PLGA-Zol at 6 weeks after surgery (Figure 1F, G), with little mineralized tissue formed around implantation tunnel with low-dose PLGA-Zol and hardly any with PLGA alone (Figure 1E, D). At week 6, average BMD and BV/TV in the metaphyseal region of femurs was higher with low-, medium- and high-dose PLGA-Zol than PLGA alone (P<0.05; Figure 2A, B). Additionally, the mean BMD was higher with both medium- and high-dose than low-dose PLGA-Zol (P<0.05; Figure 2A), with no difference between medium- and high-dose PLGA-Zol. Local controlled delivery of various doses of Zol ameliorated the pathological changes in the microstructure of bone, including increased average thickness of trabecula (P<0.05; Figure 2E) and reduced trabecular space as compared with the control (P<0.05; Figure 2D). Furthermore, trabecular thickness was greater with both medium- and high-dose than low-dose PLGA-Zol (P<0.05; Figure 2E). The trabecula number with low-dose PLGA-Zol was significantly greater than the control, medium- and high-dose PLGA-Zol treatment (P<0.05; Figure 2C).

### Medium- and high-dose PLGA-Zol improved the immature bone formation as revealed by histology

**Figure 3:**
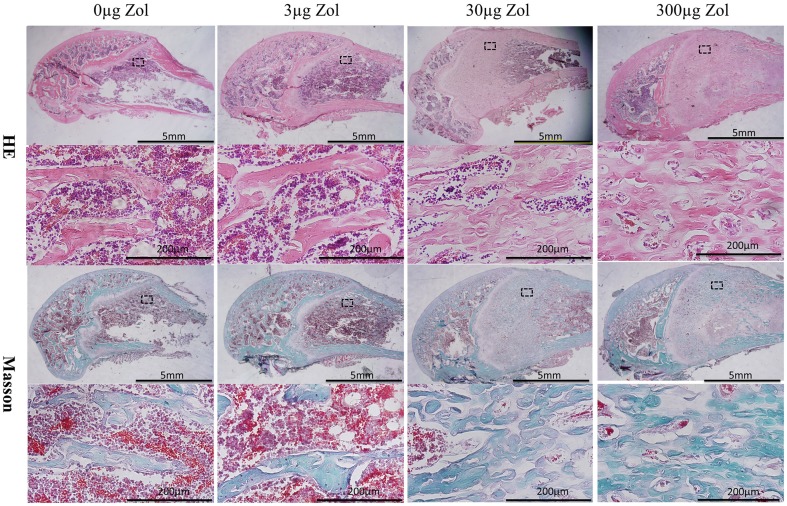
Histology of hematoxylin and eosin (HE) and Masson trichrome staining in epiphysis to metaphysis regions of specimens with PLGA-Zol and control treatment.

At 6 weeks, histology revealed a large amount of new bone formed in the region near the distal femoral epiphyseal plate with medium- and high-dose PLGA-Zol; however, unlike the trabecula in the control and low-dose groups, the structure of the new bone was irregular, defined as woven, immature bone (Figure 3). The thickness of trabecula was greater and the trabecular space was reduced with medium- and high-dose than low-dose PLGA-Zol and control treatment (Figure 3).

**Figure 4:**
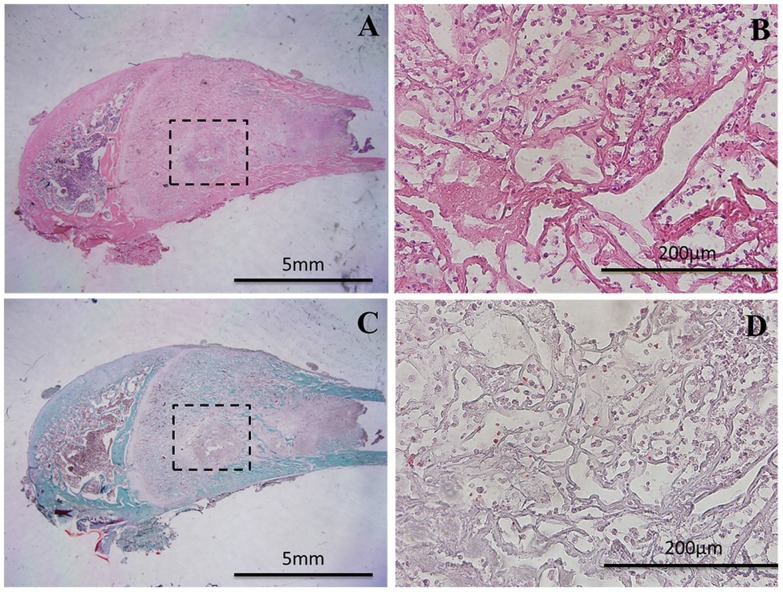
Histology of the implantation site with PLGA-30 µg Zol treatment. (A, B) Hematoxylin and eosin (HE) staining; (C, D) Masson trichrome staining.

All the PLGA and PLGA-Zol cylinders were degraded and absorbed completely, and the site of implantation was filled with a large number of bone-marrow cells and inflammatory cells. Of note, no bone formed in the centre of the implantation site in all groups (Figure 4). In addition, the length of every implanted leg is equal to the control side in all groups.

### Decreased numbers of osteoclast and increased numbers of osteoblast with PLGA-Zol delivery

**Figure 5:**
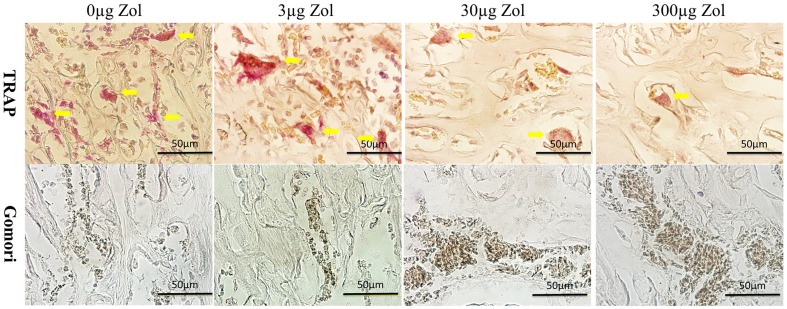
Histology of tartrate resistant acid phosphatase staining (TRAP) and alkaline phosphatase staining (ALP). Yellow arrows indicated the TRAP-positive cells in the row above and ALP-positive cells showed brown in the row below.

**Figure 6:**
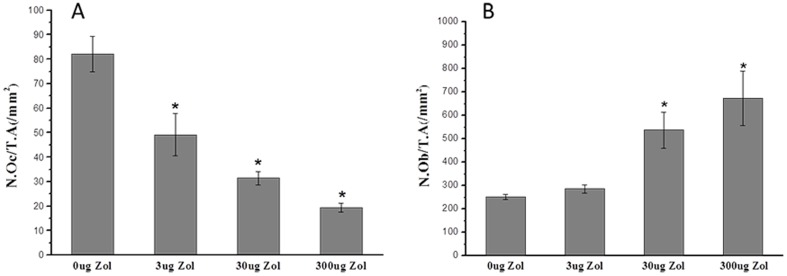
Quantitative analysis of the number of osteoclasts and osteoblasts. (A) number of osteoclasts per unit tissue area, N.Oc/T.A(/mm^2^); (B) number of osteoblasts per unit tissue area, N.Ob/T.A(/mm^2^) *P<0.05 compared with control group.

As shown in Figure 5, local controlled delivery of various doses of Zol decreased the numbers of osteoclast (TRAP-positive cells) as compared with the control. The average number of osteoclast was lower with low-, medium- and high-dose PLGA-Zol than PLGA alone (P<0.05; Figure 6A). On the other hand, medium- and high-dose PLGA-Zol increased the numbers of osteoblast (ALP-positive cells) as compared with control and low-dose PLGA-Zol. The average number of osteoblast was higher with medium- and high-dose PLGA-Zol than PLGA alone and low-dose PLGA-Zol (P<0.05; Figure 6B). However, the low-dose PLGA-Zol did not change the number of osteoblast as compared with control.

Increased expression of bone-formation markers with PLGA-Zol delivery.

**Figure 7:**
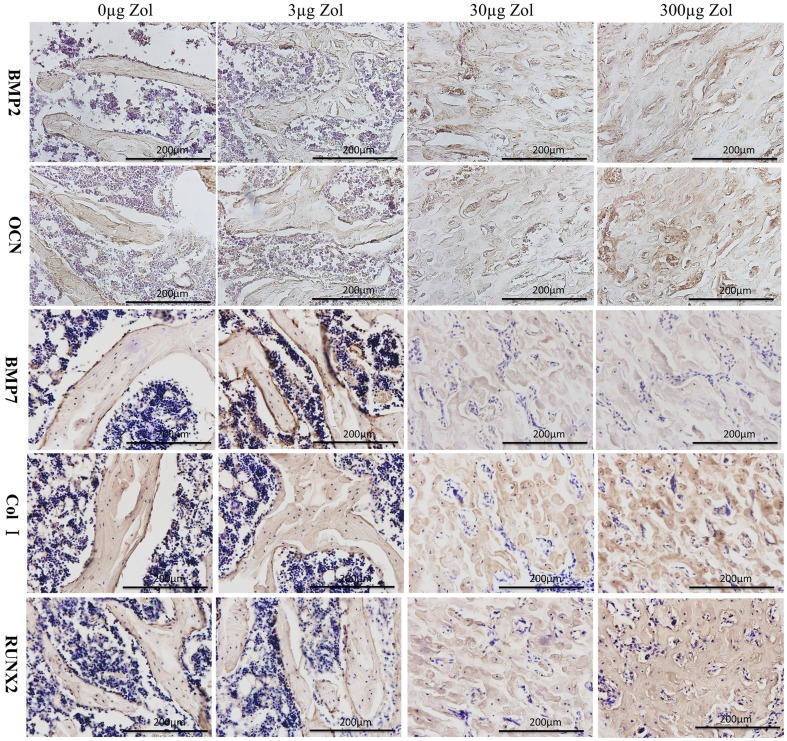
Immunohistochemistry of bone trabecular near the distal femoral epiphyseal plate. BMP2, OCN, BMP7, Col I and RUNX2 were expression in the bone matrix of newly formed trabecula. Osteocalcin (OCN), bone morphogenetic protein 2 (BMP2), bone morphogenetic protein 7 (BMP7), Type I collagen (Col I) and Runt-related transcription factor 2 (RUNX2).

**Figure 8:**
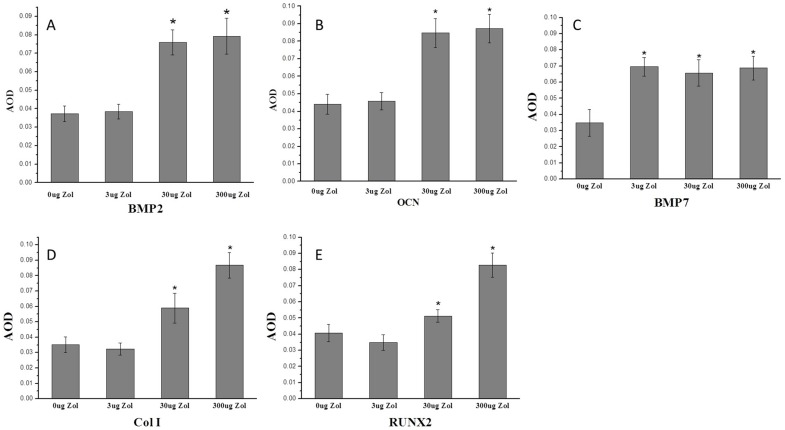
Quantitative results of immunohistochemistry staining for the expression of BMP2, OCN, BMP7, Col I and RUNX2. Osteocalcin (OCN), bone morphogenetic protein 2 (BMP2), bone morphogenetic protein 7 (BMP7), Type I collagen (Col I) and Runt-related transcription factor 2 (RUNX2). *P<0.05 compared with control group. AOD, average optical density.

To evaluate the expression of the bone-formation markers Runx2, BMP2, BMP7, OCN and Col I at 6 weeks after surgery, we used immunohistochemistry of positive stained areas (Figure 7). The AOD of BMP2, OCN, Col I and RUNX2 positive staining area was higher with medium- and high-dose PLGA-Zol than control and low-dose treatment respectively (P<0.05; Figures 8A, 8B, 8D and 8E). Furthermore, AOD of BMP7 positive staining area was higher with various doses of PLGA-Zol than PLGA alone (P<0.05; Figure 8C).

## Discussion

Our study has shown that a large amount of new bone formed near the distal femoral epiphyseal plate in rats with medium- and high-dose PLGA-Zol treatment. Controlled delivery of medium- and high-dose Zol enhanced mineralized tissue formation via decreasing the osteoclast number and in the meanwhile increasing the osteoblast number. Furthermore, medium- and high-dose PLGA-Zol accelerated the expression of bone-formation markers such as BMP2, OCN, BMP7, Col I and RUNX2 at the site of implantation.

Therapeutic approaches with BPs represent a convenient and effective mode of treating bone-related cancers, Paget's disease, and especially osteoporosis [23]. Intravenous Zol within 90 days after repair of low-trauma hip fracture was associated with the reduced rate of new clinical fractures and improved survival [24]. However, BPs inhibiting bone resorption cannot completely explain the reduction in fracture incidence. Most research has focused on inhibiting bone resorption by targeting the end effector cell, the osteoclast. Recently, some *in vitro* and *in vivo* evidence indicated that BPs may promote osteoblastic bone formation [9], [11], [25]. On the contrary, others suggested that BPs could decrease the proliferation of and inhibit osteoblast differentiation and mineralization [26]–[28]. Some researchers suggested that growth and prodifferentiation effects are exerted at low concentrations of BPs, with the inhibitory effects exerted at high concentrations [1]. Additionally, the effects of BPs may be time-dependent [29]. Thus, in this study, we used PLGA as a drug carrier to deliver the BP Zol and investigated the effect on local bone formation in rat. Controlled delivery of medium- and high-dose Zol enhanced bone formation at the site of implantation. In our study, the average BMD and BV/TV of the femoral metaphysis were greater with medium- and high-dose than low-dose PLGA-Zol and control treatment. The trabecular thickness was greater and the trabecular space was reduced with medium- and high-dose than low-dose PLGA-Zol and control treatment. However, the structure of the new bone was irregular. Using PLGA as a drug carrier for controlled delivery of Zol may promote local bone formation.

Some researchers investigated the effect of local delivery of BPs from screw or titanium implants *in vivo* and also found that locally delivered BPs could improve the bone density around the implant. However, biomechanical analysis showed that the maximal pullout force of the implant was associated with BPs concentration and treatment time [30], [31].

The mechanism by which BPs increase bone mineral density and enhance bone formation is not completely understood. In the cellular level, many studies have shown that BPs can affect osteoclast recruitment, differentiation, and resorptive activity, and some may induce apoptosis [32]. On the other hand, BPs are able to prevent osteoblast apoptosis and have an anabolic effect on osteoblasts, which is an important role in enhancing bone formation [10], [33]. The decreased osteoclast number and increased osteoblast number in our study also demonstrated this point. Someone suggested that the anti-apoptotic effects of BPs do not depend on inhibitory effects on osteoclasts because analogs that lack anti-resorptive activity could still inhibit apoptosis in osteoblasts without decreasing osteoclast viability [34], [35]. BPs' anabolic effect on osteoblast might be via upregulation of BMP2, which is well recognized for stimulating proliferation and differentiation of mesenchymal progenitor cells.Thus resulting in the production of bone tissue [36]. It has been demonstrated that BMP2 is a necessary component of the signaling cascade that governs fracture repair [37]. In our study, immunohistochemistry revealed greater expression of BMP2 with medium- and high-dose than low-dose PLGA-Zol and control treatment. This result is consistent with the mass of newly formed bone. Other studies indicated that the anabolic effect of BPs on osteoblasts was possibly mediated by Cx43 hemichannel opening, thus leading to activation of Src/ERK [35], [38], [39]. Also, the combined use of BMP2 and Zol could enhance bone fracture healing in a mouse model of neurofibromatosis type 1 deficiency [40].

OCN is described as a necessary protein for bone mineralization. Our results show that local controlled delivery of low-dose Zol did not affect its expression, whereas medium- and high-dose PLGA-Zol increased its expression. In agreement with our results, Ponader et al. found a positive differentiation stimulus on osteoblast differentiation with quantification of alkaline phosphatase and OCN gene expression [41]. Koch et al. found that Zol at high concentrations enhanced the gene expression of OCN, whereas lower concentrations led to decreased gene expression [42].

The present study is potentially limited by the fact that detecting the distribution of local controlled delivery of Zol *in vivo* is difficult. The exact drug concentration on the site of implantation at different times is not clear and the mechanism of local action at various concentrations is not completely understood. In addition, the end time of this experiment was 6 weeks after surgery, which may be not the best observation period for new bone formation induced by BPs, because some investigators suggested that BPs could reduce bone turnover rate [43], [44].

## Conclusions

Our study supports that using PLGA as a drug carrier to deliver the BP Zol could promote local bone formation in rat. This method could be used for promoting local bone formation in the femoral head of osteonecrosis. However, this hypothesis needs further testing before clinical validation and further well-designed studies.
